# Relationship Between Subclinical Renal Damage and Maximum Rate of Blood Pressure Variation Assessed by Fourier Analysis of 24-h Blood Pressure Curve in Patients with Essential Hypertension

**DOI:** 10.3390/life15071149

**Published:** 2025-07-21

**Authors:** Caterina Carollo, Alessandra Sorce, Maria Giovanna Vario, Emanuele Cirafici, Davide Bologna, Maria Elena Ciuppa, Salvatore Evola, Guseppe Mulè, Giulio Geraci

**Affiliations:** 1 Unit of Nephrology and Dialysis, Hypertension Excellence Centre, Department of Health Promotion, Mother and Child Care, Internal Medicine and Medical Specialties (PROMISE), University of Palermo, 90133 Palermo, Italy; alessandra.sorce@community.unipa.it (A.S.); mariagiovanna.vario@policlinico.pa.it (M.G.V.); emanuele.cirafici@community.unipa.it (E.C.); davide.bologna@community.unipa.it (D.B.); giuseppe.mule@unipa.it (G.M.); 2 Department of Health Promotion, Mother and Child Care, Internal Medicine and Medical Specialties, University of Palermo, 90133 Palermo, Italy; mariaelena.ciuppa@community.unipa.it (M.E.C.); salvatore.evola@policlinico.pa.it (S.E.); 3 Department of Medicine and Surgery, “Kore” University of Enna, 94100 Enna, Italy; giulio.geraci@unikore.it

**Keywords:** hypertension, ABPM, kidney damage, BPV, Fourier analysis, subclinical renal damage

## Abstract

Background: Blood pressure (BP) variability has been increasingly recognized as a predictor of cardiovascular and renal outcomes. However, the relevance of specific dynamic indices such as the maximum slope of systolic blood pressure (max SBP slope), derived through partial Fourier series modeling, in relation to early renal damage remains underexplored. Methods: A total of 389 patients with essential hypertension were enrolled and stratified according to the estimated glomerular filtration rate (eGFR) ≥ or <90 mL/min/1.73 m^2^ and the presence of subclinical renal damage, defined by elevated urinary albumin excretion (AER) and/or reduced eGFR. All participants underwent clinical and biochemical evaluation, as well as 24-h ambulatory blood pressure monitoring (ABPM), including advanced hemodynamic analysis using Fourier-based modeling. Results: Patients with eGFR < 90 mL/min/1.73 m^2^ were older and exhibited higher waist circumference, uricemia, albuminuria, and systolic BP values, including the elevated max SBP slope (12.8 vs. 10.8 mmHg/h, *p* = 0.028). Subclinical renal damage was associated with older age; male sex; smoking; and higher levels of uricemia, clinical, and ambulatory BP, and the max SBP slope (14.2 vs. 10.7 mmHg/h, *p* = 0.007). The max SBP slope positively correlated with AER (*r* = 0.215, *p* < 0.001) and inversely with eGFR (*r* = −0.153, *p* = 0.002). In multivariate linear regression, the max SBP slope remained independently associated with AER (*β* = 0.220, *p* < 0.001), along with mean 24-h SBP, male sex, and the day–night SBP percentage dip. Logistic regression confirmed these associations with subclinical renal damage (max SBP slope OR: 1.536; 95% CI: 1.241–2.004; *p* = 0.001). Conclusions: The max SBP slope, a dynamic index of BP derived via Fourier analysis, is independently associated with markers of subclinical renal damage in hypertensive patients. This suggests that incorporating such advanced metrics into ABPM evaluation may improve early risk stratification and help identify individuals at greater risk of renal impairment, even in the absence of overt kidney disease.

## 1. Introduction

Essential hypertension (EH) is one of the most prevalent chronic conditions worldwide and a leading cause of both cardiovascular disease and chronic kidney disease (CKD) [1,2]. While elevated mean blood pressure (BP) values have traditionally been used for risk stratification [3,4], growing evidence suggests that blood pressure variability (BPV)—particularly short-term variability assessed over a 24-h period—may provide additional and clinically significant information regarding end-organ damage [5,6,7]. In this context, subclinical renal damage, often preceding overt CKD, represents a critical and potentially reversible target of early intervention.

Subclinical kidney injury in hypertensive individuals is frequently characterized by either a mild-to-moderate reduction in estimated glomerular filtration rate (eGFR) or the presence of microalbuminuria, both of which are associated with adverse renal and cardiovascular outcomes [8,9,10]. Importantly, this type of damage often occurs in the absence of sustained elevations in BP, underscoring the importance of additional hemodynamic factors in its pathogenesis. Among these, the dynamic properties of BP—particularly its temporal fluctuations and rapid transitions—may exert mechanical and functional stress on the renal microcirculation, thereby contributing to glomerular and tubular injury [11,12].

Methodological advances in the analysis of BP signals have facilitated a more detailed characterization of BP dynamics. In particular, the application of mathematical techniques such as Fourier Analysis to 24-h ambulatory blood pressure monitoring (ABPM) allows for the decomposition of the BP signal into its constituent frequency components. This approach enables the quantification of cyclical patterns and, importantly, the estimation of the maximum rate of pressure change (Max dP/dt), a parameter reflecting the steepest instantaneous slope of the BP curve within a circadian cycle [13].

Recent advances in blood pressure monitoring techniques, including ambulatory blood pressure monitoring (ABPM) and self-blood pressure monitoring (SBPM), have improved the ability to capture dynamic changes and variability in blood pressure outside clinical settings. These tools not only enhance hypertension awareness and treatment adherence but also provide valuable insights into blood pressure variability patterns that traditional office measurements may miss [14,15,16]. Such comprehensive monitoring is crucial for identifying early hemodynamic stress that may lead to subclinical renal damage.

Despite its potential, the application of Fourier analysis to ABPM data remains relatively uncommon in both clinical practice and cardiovascular research. Most studies continue to rely on conventional indices of BPV, such as standard deviation (SD), average real variability (ARV), and weighted SD (wSD), which, although straightforward, often fail to capture the speed and complexity of BP changes. Fourier-based approaches provide a more continuous and noise-resistant representation of pressure fluctuations, allowing for the extraction of physiologically relevant features such as the rate of BP variation. The integration of such dynamic metrics into clinical research may uncover previously overlooked associations between hemodynamic instability and early organ damage—particularly in vulnerable vascular beds such as the renal microcirculation.

Several studies have shown that increased short-term BPV is associated with greater arterial stiffness, endothelial dysfunction, and early target organ damage, including left ventricular hypertrophy and silent cerebrovascular lesions [17,18,19,20]. However, the association between rapid BP fluctuations—quantified through Fourier-based time-derivative metrics—and subclinical renal damage remains underexplored. Specifically, it is not yet established whether the maximum rate of systolic or diastolic pressure change (as derived from Fourier-transformed ABPM data) correlates with early markers of renal impairment in untreated or newly diagnosed hypertensive individuals.

Given the kidneys’ unique vulnerability to hemodynamic stress—owing to their low vascular resistance, high perfusion, and autoregulatory constraints—it is biologically plausible that exaggerated and frequent oscillations in BP could contribute to renal microvascular injury, even in the absence of sustained hypertension. Furthermore, such variations may lead to cyclical ischemia-reperfusion injury, podocyte stress, and glomerular hyperfiltration, ultimately manifesting as microalbuminuria or subtle declines in eGFR [21,22].

Therefore, the aim of the present study is to investigate the relationship between subclinical renal damage—defined by eGFR between 30 and 60 mL/min/1.73 m^2^ and/or the presence of microalbuminuria—and the maximum rate of change of blood pressure (systolic and diastolic) over 24 h, calculated via the partial Fourier series fitting of ABPM data, in a cohort of untreated essential hypertensive patients. We hypothesize that individuals exhibiting steeper BP slopes—indicative of more abrupt pressure transitions—are more likely to present with early renal injury, independent of mean 24-h BP values and conventional BPV metrics.

By elucidating this relationship, the study may provide novel insights into the pathophysiology of hypertensive nephropathy and support the incorporation of frequency-domain BP analysis into clinical risk assessment models for early renal damage. Moreover, these findings could have implications for the development of targeted antihypertensive strategies aimed at not only reducing mean BP but also attenuating its most damaging temporal components.

## 2. Materials and Methods

### 2.1. Study Population

Patients were consecutively recruited from those referred to our regional hypertension referral center. All participants underwent a thorough medical history, physical examination, and diagnostic work-up to exclude cases of secondary hypertension. For patients already on antihypertensive therapy, evaluations were performed after a minimum washout period of seven days. The following exclusion criteria were applied:

- History of renovascular, parenchymal, endocrine, or malignant hypertension;
- Presence of hematuria or overt proteinuria;
- Personal history of glomerulonephritis or hereditary kidney disease;
- Errors in 24-h urine collection, defined as:
  ○ Under-collection: urinary creatinine < 10 mg/kg for women or <15 mg/kg for men;
  ○ Over-collection: urinary creatinine > 25 mg/kg for women or >30 mg/kg for men;
- Inability to obtain at least 80% valid BP readings during 24-h ambulatory blood pressure monitoring (ABPM);
- History or clinical signs of heart failure, ischemic heart disease, or cerebrovascular disease;
- Presence of major non-cardiovascular comorbidities;
- Estimated glomerular filtration rate (eGFR) < 30 mL/min/1.73 m^2^;
- Inability to suspend antihypertensive medications for at least one week prior to ABPM and biochemical assessment.

After applying the exclusion criteria, a total of 389 patients with essential hypertension were enrolled in the study. All participants provided written informed consent prior to inclusion. The diagnosis of hypertension was established according to the 2023 guidelines of the European Society of Hypertension (ESH) and the European Society of Cardiology (ESC). Office blood pressure (BP) was defined as the average of three measurements obtained at 5-min intervals with the patient seated.

### 2.2. Definition of Subclinical Kidney Damage

Subclinical renal damage was defined as the presence of an albumin excretion rate (AER) between 20 and 200 μg/min in patients with an estimated glomerular filtration rate (eGFR) greater than 60 mL/min/1.73 m^2^.

### 2.3. Laboratory Methods

Routine biochemical parameters were measured using standard automated techniques. The glomerular filtration rate was estimated using the CKD-EPI 2009 creatinine-based equation. The albumin excretion rate in 24-h urine samples was measured via radioimmunoassay (Techno Genetics RIA Kit). Microalbuminuria was defined as AER > 20 μg/min and <200 μg/min.

### 2.4. Ambulatory Blood Pressure Monitoring (ABPM)

Twenty-four-hour ABPM was performed using a non-invasive portable device (Spacelabs 90207, Redmond, WA, USA). BP was measured every 15 min during daytime (7:00–22:00) and every 20 min at night (22:00–7:00). Data were reviewed using the Spacelabs ABP90209 interface (v. 2.40.23). Readings were excluded if:

- SBP > 260 mmHg or <70 mmHg;
- DBP > 150 mmHg or <40 mmHg;
- Pulse pressure > 150 mmHg or <20 mmHg.

Data were processed using CHRONOS ABPM-FIT software for Fourier analysis.

### 2.5. Fourier-Derived Parameters

The following parameters were extracted:

1. The maximum slope of SBP and DBP (Slope max SBP and DBP), calculated as the first derivative of the Fourier-fitted curve in mmHg/hour.
2. The maximum and minimum BP values (SBP max/min and DBP max/min), and their absolute differences.
3. The weighted standard deviation (wSD) of 24-h SBP and DBP, derived from day and night SDs.

### 2.6. Circadian BP Variability

Circadian variability was calculated as:

(Daytime BP − Nighttime BP) × 100/Daytime BP

Nocturnal dipping status was classified as:

- Reverse dippers: nighttime BP > daytime BP
- Non-dippers: night–day difference 0–10%
- Dippers: night–day difference 10–20%
- Extreme dippers: reduction > 20%

### 2.7. Fourier Analysis

To model circadian BP variation, Fourier analysis was used:

PA(t) = M + A_1_·cos((2π·t/24) − φ_1_) + A_2_·cos((4π·t/24) − φ_2_) + … + Aₖ·cos((2π·K·t/24) − φₖ)

where PA(t) is the BP (systolic or diastolic) at time t, M is the MESOR (midline estimating statistic of rhythm), Aₖ is the amplitude of the k-th harmonic, φₖ is the acrophase (timing of the peak of the harmonic).

The first harmonic represents a 24-h cycle, the second 12-h, the third 8-h, and so on. The model fit was evaluated using R^2^ (the coefficient of determination). Compared to Single Cosinor analysis, Fourier analysis allows for better curve fitting with added harmonics.

Advantages include artifact resilience, improved peak/trough estimates, and slope calculations (i.e., the maximum BP rate of rise). The slope is theoretically analogous to the Time Rate Index and the morning BP surge, which has been associated with adverse cardiovascular outcomes.

### 2.8. Statistical Analysis

All continuous variables were tested for normality using the Kolmogorov–Smirnov test. Variables that did not follow a Gaussian distribution included urinary albumin excretion, triglycerides, BP max–min difference, Slope max BP, and weighted standard deviation of BP (wSD BP). These variables were log-transformed using base 10 prior to statistical analysis and are reported as medians and interquartile ranges (25th–75th percentiles). Normally distributed continuous variables are reported as mean ± standard deviation.

The study population was stratified into groups based on eGFR above or below 90 mL/min/1.73 m^2^ and the presence or absence of subclinical kidney damage, defined according to the 2024 ESC/ESH guidelines.

Group differences in continuous variables were assessed using the independent samples t-test, while categorical variables were compared using the χ^2^ test with Yates’ correction. Associations between variables were evaluated using simple linear regression, Pearson’s correlation coefficients, and stepwise multiple linear regression. In the latter, eGFR and AER were considered as dependent variables in separate models, while explanatory variables included those significantly associated with eGFR in univariate analysis and those selected based on biological or pathophysiological plausibility.

Additionally, a stepwise multiple logistic regression analysis was conducted using the presence of subclinical kidney damage as the dependent variable. The same covariates used in the linear regression models were included as independent variables. All continuous predictors were standardized prior to entry into the model, so that their effects were expressed per standard deviation change.

A *p*-value < 0.05 was considered statistically significant.

All analyses were performed using IBM SPSS Statistics for Windows, Version 26.0 (IBM Corp., Armonk, NY, USA).

## 3. Results

Table 1 presents the demographic and clinical characteristics of the overall study population and of patients stratified according to estimated glomerular filtration rate (eGFR) above or below 90 mL/min/1.73 m^2^.

Compared to patients with preserved renal function, patients with reduced eGFR were significantly older (50.7 ± 13.6 vs. 47.9 ± 11.8 years, *p* = 0.04), had greater waist circumference (98.6 ± 14.7 vs. 95.8 ± 11.1 cm, *p* = 0.04), higher serum uric acid levels (5.2 ± 1.5 vs. 4.7 ± 1.4 mg/dL, *p* = 0.001), and higher albuminuria (10 [5–31] vs. 8 [5–17] mg/min, *p* < 0.001).

A borderline difference was observed for triglycerides (140 [107–202.5] vs. 130 [88–180] mg/dL, *p* = 0.0549). No significant differences were found between groups in sex distribution, smoking status, diabetes prevalence, glycemia, BMI, lipidic profile, or prior antihypertensive therapy (>0.05). Systolic blood pressure (SBP) was significantly higher in the lower eGFR group (136 ± 13.4 vs. 130 ± 12.5 mmHg, *p* < 0.001), while the difference in diastolic blood pressure (DBP) did not reach statistical significance (84 ± 11.1 vs. 80 ± 11.7 mmHg, *p* = 0.09).

Table 2 presents the blood pressure parameters obtained through 24-h ambulatory blood pressure monitoring in subjects stratified by estimated glomerular filtration rate (eGFR) above or below 90 mL/min/1.73 m^2^.

Patients with eGFR < 90 mL/min/1.73 m^2^ exhibited significantly higher 24-h, daytime, and nighttime mean systolic blood pressure (SBP) compared to those with eGFR > 90 mL/min/1.73 m^2^ (all *p* < 0.001). Nighttime mean diastolic blood pressure (DBP) was also significantly elevated in the lower eGFR group (*p* = 0.001), while 24-h and daytime DBP values did not differ significantly. Among the blood pressure variability indices, the Slope max of SBP was significantly higher in subjects with eGFR < 90 mL/min/1.73 m^2^ (*p* = 0.028), whereas other parameters such as SBP and DBP maxima, SBP and DBP ranges, and the Slope max of DBP showed no significant differences between groups.

An additional stratification of the study population was performed based on the presence or absence of subclinical renal damage. As shown in Table 3, patients with subclinical renal damage were older compared to those without, and more frequently male and smokers. This group also exhibited higher levels of serum uric acid, urinary albumin excretion, and both clinical systolic (SBP) and diastolic blood pressure (DBP).

Furthermore, as reported in Table 4, these patients presented with higher 24-h, daytime, and nighttime SBP and DBP values, as well as higher maximum SBP and DBP, and a steeper maximum SBP slope, compared to those without subclinical renal impairment. No significant differences were observed between the two groups for any other parameters. In particular, no statistically significant differences were found in the 24-h blood pressure variability (weighted standard deviation, WSD) of either SBP (*p* = 0.57) or DBP (*p* = 0.71).

**Figure 1:**
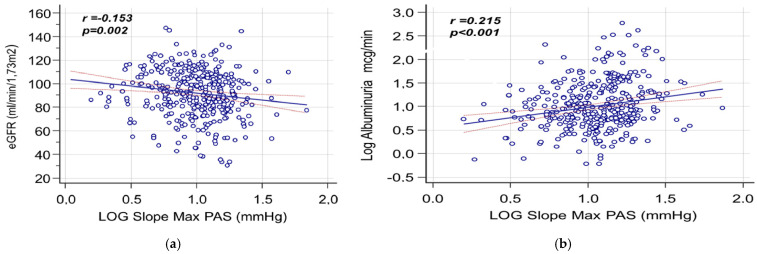
(**a**) Univariate Pearson correlation coefficients between eGFR and maximum slope of SBP. (**b**) Univariate Pearson correlation coefficients between AER and maximum slope of SBP.

Table 5 shows the univariate Pearson correlation coefficients between eGFR, AER, and hemodynamic parameters obtained through ABPM. Figure 1a,b present regression lines depicting the relationships between the maximum slope of SBP and, respectively, eGFR and AER.

**Figure 2:**
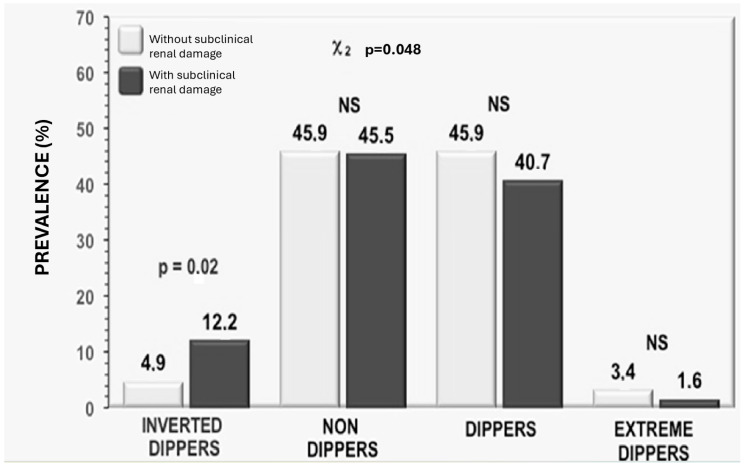
Prevalence of the different patterns of circadian systolic blood pressure variability in the study population, classified according to the magnitude of the percentage day–night SBP decline, in subjects with (black bars) and without (grey bars) subclinical kidney damage.

The evaluation of the circadian rhythm of systolic and diastolic blood pressure showed that the percentage day–night difference (Δ% day–night) in SBP, but not in DBP, was significantly lower in subjects with subclinical renal damage (*p* = 0.02) compared to patients without such damage. No significant differences in this parameter were found between subjects with eGFR below or above 90 mL/min/1.73 m^2^. Categorizing subjects as “dippers” or “non-dippers” and comparing these groups among the studied cohorts revealed no significant differences within the subgroups: with eGFR> or <90 mL/min/1.73 m^2^, with or without subclinical renal damage. Conversely, when a more detailed classification of patients was applied based on the circadian blood pressure pattern into “dippers,” “non-dippers,” “extreme dippers,” and “inverted dippers,” only the latter category—and limited to SBP—was found to be more frequently present in subjects with subclinical renal damage (Figure 2) compared to those with normal AER and hypertensive patients without renal morpho-functional alterations (Figure 2).

Only the association between AER and max SBP slope remained significant in multivariate analyses (Table 6), where AER and eGFR were alternatively considered as dependent variables. In these multiple linear regression models, the following potential explanatory variables were included: mean 24-h SBP, sex, age, smoking status, diabetes, prior antihypertensive therapy, blood glucose, and waist circumference (or BMI). The statistical significance of this association persisted even after adding other short-term blood pressure variability indices into the multivariate model, such as the difference between maximum and minimum SBP values estimated through the Fourier analysis of the 24-h blood pressure curve, the weighted standard deviation of 24-h blood pressure, and the percentage day–night SBP decline.

Of all these covariates, those independently associated with AER—besides max SBP slope—were mean 24-h SBP, sex, and the day–night SBP delta, while the only variables independently correlated with eGFR were age and mean 24-h SBP.

In multiple logistic regression analysis (Table 7), where subclinical renal damage was the dependent variable, the same parameters independently associated with AER in the multiple linear regression analysis were found to be significantly associated.

## 4. Discussion

The present study investigates the association between dynamic features of ambulatory blood pressure monitoring (ABPM) profiles and early markers of renal damage, focusing on the steepest systolic blood pressure (SBP) increases extracted using a partial Fourier series model. Our findings support the hypothesis that slope-derived indices, obtained via frequency-domain modeling rather than conventional time-domain metrics, may provide novel insights into short-term blood pressure variability (BPV) and its role in early renal injury.

Subclinical renal damage—defined by microalbuminuria or reduced eGFR in the absence of overt kidney disease—was associated with older age, male sex, smoking, and elevated serum uric acid, all established cardiovascular risk factors. These patients also exhibited higher office and ambulatory SBP, including nocturnal values, highlighting the burden of uncontrolled hypertension. Notably, the maximum SBP slope, reflecting the steepest increase in SBP over 24 h, was significantly higher in patients with subclinical renal damage and remained independently associated with albumin excretion rate (AER) after adjusting for age, sex, average 24-h SBP, and circadian BP decline.

This finding is pathophysiologically relevant given that rapid, transient BP surges impose mechanical stress on the vascular endothelium and renal microcirculation. Such abrupt increases may lead to glomerular hypertension, endothelial dysfunction, and albuminuria—a key marker and mediator of renal injury. The independent association between SBP slope and both AER and subclinical renal damage suggests that short-term BP instability, rather than sustained BP elevation alone, plays a critical role in early renal impairment.

Our results also confirm the well-documented dissociation between markers of early glomerular damage (e.g., albuminuria) and overall renal function (e.g., eGFR). In line with prior studies, the SBP slope showed a stronger correlation with albuminuria than with eGFR, which often remains preserved in early disease stages. This supports albuminuria as a more sensitive early marker, while slope-based metrics may serve as early indicators of progressive renal risk before measurable functional decline occurs [23,24].

Unlike traditional BPV measures that reflect average levels or overall variability—such as standard deviation (SD), weighted SD (wSD), or average real variability (ARV)—the SBP slope captures the most rapid increases in systolic pressure within the 24-h profile. These fast dynamics, often missed by time-domain analyses, reflect sharp hemodynamic changes and vascular stress relevant to renal damage.

While evidence linking BPV to subclinical organ damage is growing, a significant debate remains. Many studies report associations between elevated short-term BPV (e.g., SD, ARV) and early vascular or renal damage—including increased carotid intima-media thickness, left ventricular hypertrophy, and microalbuminuria—but the results are inconsistent [25,26,27,28,29]. These discrepancies stem from differences in BPV metrics, measurement intervals, and patient populations [30,31]. Traditional BPV indices also have limitations. SD conflates circadian [25,26,27,28,29] variation with pathological fluctuations, ARV lacks information on rate of change, and wSD depends heavily on measurement timing. In contrast, the SBP slope emphasizes the first derivative of SBP—highlighting brief, potentially harmful BP surges that may not affect mean BP or conventional variability metrics. Its accuracy, however, depends on high-resolution data, requiring frequent BP sampling (e.g., every 5 min or less) [32,33,34].

From a physiological standpoint, this is crucial: the kidney’s glomerular network is highly sensitive to both sustained and pulsatile hemodynamic forces. Steep, sudden BP increases can damage the glomerular filtration barrier, triggering endothelial injury, increased permeability, and albuminuria—even in patients with otherwise normal BP profiles [35].

The Fourier-based modeling approach allows the 24-h BP curve to be reconstructed as a smooth, continuous signal composed of sinusoidal components. Within this fitted curve, the maximum SBP slope corresponds to the point of greatest steepness—the highest first derivative—offering a new perspective on BPV. While Fourier analysis has traditionally been used to study circadian BP rhythms (e.g., mesor, amplitude, and acrophase [36,37]), the extraction of the maximum SBP slope represents an innovative application of this method.

## 5. Conclusions

Our findings suggest that frequency-domain-based dynamic metrics provide additional pathophysiological insight and may serve as early markers of renal injury. Incorporating such slope-derived indices into ambulatory blood pressure monitoring analysis could enhance risk stratification and facilitate the earlier identification of patients at risk for progressive kidney damage, even in the absence of sustained hypertension or overt renal dysfunction.

## Figures and Tables

**Table 1:** Characteristics of the study population stratified by eGFR above or below 90 mL/min/1.73 m^2^.

	Total Patients	GFR > 90 mL/min/1.73 m^2^	eGFR < 90 mL/min/1.73 m^2^	*p*
	N = 389	N = 266	N = 123	
Age (years)	48.8 ± 12.4	47.9 ± 11.8	50.7 ± 13.6	0.04
Sex (Males) (%)	57.8	58	57.7	0.51
Smokers (%)	36.7	33.5	40.2	0.16
Diabetes (%)	11.2	9.1	13.4	0.12
Glycemia (mg/dL)	99.4 ± 24.4	98.2 ± 20.7	102.2 ± 31.2	0.15
BMI (kg/m^2^)	28.4 ± 4.1	28.2 ± 4.2	28.8 ± 4.1	0.16
Waist circumference (cm)	96.7 ± 12.4	95.8 ± 11.1	98.6 ± 14.7	0.04
Total cholesterol (mg/dL)	211.6 ± 40.7	211.5 ± 41.2	211.8 ± 39.7	0.95
HDL cholesterol (mg/dL)	45.4 ± 9.8	46.3 ± 9.5	43.5 ± 10.5	0.34
Triglycerides (mg/dL)	134 (94–189)	130 (88–180)	140 (107–202.5)	0.0549
Uricaemia (mg/dL)	4.9 ± 1.5	4.7 ± 1.4	5.2 ± 1.5	0.001
Albuminuria (mg/min)	9 (5–22)	8 (5–17)	10 (5–31)	<0.001
Previous antihypertensive therapy (%)	72.6	69.6	75.9	0.11
Office SBP (mmHg)	154 ± 19	130 ± 12.5	136 ± 13.4	<0.001
Office DBP (mmHg)	93 ± 18	80 ± 11.7	84 ± 11.1	0.09

**Table 2:** Ambulatory blood pressure parameters stratified by estimated glomerular filtration rate (eGFR) above or below 90 mL/min/1.73 m^2^.

	Total Patients	eGFR > 90 mL/min	eGFR < 90 mL/min	*p*
	N = 389	N = 266	N = 123	
24 h SBP (mmHg)	132 ± 13.2	130 ± 13.5	136 ± 13.2	<0.001
24 h DBP (mmHg)	81.7 ± 11.6	81 ± 11.7	84 ± 11.1	0.06
daytime SBP(mmHg)	136 ± 13.5	134 ± 13.03	140 ± 13.8	<0.001
daytime DBP (mmHg)	86 ± 11	85 ± 10.8	87 ± 11.5	0.27
nocturnal SBP (mmHg)	123 ± 14.3	120 ± 15	129 ± 14.3	<0.001
nocturnal DBP (mmHg)	74 ± 11	73 ± 10.5	76 ± 11.5	0.001
SBP max (mmHg)	150 (140–162)	148 (139–161)	153 (141–165)	0.14
DBP max (mmHg)	98 (89–105)	98 (90–106)	97 (88–105)	0.58
SBP max–min (mmHg)	37 (28–46)	36 (27–44)	39 (28–48)	0.102
DBP max–min (mmHg)	32 (25–39)	32 (25–39)	31 (25–37)	0.487
Slope max SBP	11.7 (7.9–16.5)	10.8 (7.6–15.1)	12.8 (8.9–17.6)	0.028
Slope max DBP	10.6 (7.9–14.3)	10.7 (7.9–14.2)	10.5 (7.8–14.3)	0.736

**Table 3:** Clinical and biochemical characteristics of patients stratified by the presence or absence of subclinical renal damage.

	Patients Without Subclinical Renal Damage N = 272	Patients with Subclinical Renal Damage N = 117	*p*
Age (years)	48 ± 11.7	51 ± 13.8	0.03
Sex (Males) (%)	53.7	68.3	0.008
Smokers (%)	32.8	46.4	0.019
Diabetes (%)	10.3	13.3	0.512
Glycemia (mg/dL)	99 ± 22.1	101 ± 30	0.41
BMI (kg/m^2^)	28.2 ± 4.2	28.8 ± 4.1	0.23
Waist circumference (cm)	95.9 ± 11.2	98.4 ± 14.9	0.08
Total cholesterol (mg/dL)	212 ± 41	210.5 ± 49.7	0.73
HDL cholesterol (mg/dL)	46.1 ± 9.4	43.7 ± 10.8	0.07
Triglycerides (mg/dL)	130 (93–182)	152 (99–200)	0.08
Uricaemia (mg/dL)	4.7 ± 1.4	5.3 ± 1.6	0.001
AER (μg/min)	6 (4–10)	23 (37–57)	<0.001
Previous antihypertensive therapy (%)	70.5	77.8	0.167
Clinical SBP (mmHg)	152 ± 18	159 ± 19	0.001
Clinical DBP (mmHg)	92 ± 17	96 ± 19	0.043

**Table 4:** Comparison of blood pressure values obtained by ambulatory blood pressure monitoring (ABPM) between patients without subclinical renal damage and patients with subclinical renal damage.

	Patients Without Subclinical Renal Damage	Patients With Subclinical Renal Damage	*p*
	N = 272	N = 117	
24 h SBP (mmHg)	130 ± 13	137 ± 13	<0.001
24 h DBP (mmHg)	81 ± 12	84 ± 11	0.008
daytime SBP(mmHg)	134 ± 13	140 ± 14	<0.001
daytime DBP (mmHg)	85 ± 11	87 ± 11	0.037
nocturnal SBP (mmHg)	121 ± 13	129 ± 15	0.001
nocturnal DBP (mmHg)	73 ± 11	77 ± 12	0.002
SBP max (mmHg)	147 (138–160)	157 (144–165)	0.01
DBP max (mmHg)	96 (89–105)	100 (91–108)	0.054
SBP max–min (mmHg)	11 (7–15)	14 (10–18)	0.014
DBP max–min (mmHg)	10 (8–14)	11 (8–15)	0.164
Slope max SBP (mmHg/h)	10.7 (7.6–15.5)	14.2 (10.2–18.2)	0.007
Slope max DBP (mmHg/h)	10.3 (7.0–13.9)	11.4 (7.9–14.8)	0.92

**Table 5:** Univariate Pearson correlation coefficients between eGFR, AER, and hemodynamic parameters obtained through ABPM.

	GFR		Log MA	
	r	*p*	r	*p*
24 h SBP	−0.198	<0.001	0.279	<0.001
24 h DBP	−0.091	ns	0.189	<0.001
daytime SBP	−0.179	<0.001	0.249	<0.001
daytime DBP	−0.09	ns	0.158	0.002
nocturnal SBP	−0.21	<0.001	0.298	<0.001
nocturnal DBP	−0.118	0.02	0.187	<0.001
(Log) Slope max SBP	−0.153	0.002	0.215	<0.001
(Log) Slope max DBP	−0.008	ns	0.138	0.007
(Log) SBP max	−0.145	0.005	0.259	<0.001
(Log) DBP max	−0.005	ns	0.192	<0.001
(Log) SBP max–min	−0.127	0.008	0.156	0.002
(Log) DBP max–min	0.025	ns	0.152	0.003
Δ% day–night SBP	0.09	0.075	−0.114	0.025

**Table 6:** Multivariate linear regression models for (a) log-transformed urinary albumin excretion and (b) estimated glomerular filtration rate (eGFR).

(a)	(Log) Urinary Albumin Excretion (R^2^ = 0.154)	
	β	*p*
24 h average SBP	0.231	<0.001
(Log) Slope max SBP	0.220	<0.001
Sex (M: 1; F = 0)	0.152	0.001
Δ% day–night SBP	−0.142	0.003
(b)	eGFR (R^2^ = 0.243)	
	β	*p*
Age	−0.453	<0.001
24 h average SBP	−0.152	0.001

**Table 7:** Multivariate logistic regression model for subclinical renal damage.

	Subclinical Renal Damage (R^2^ = 0.154)		
	Odds ratio	95% CI	*p*
24 h average SBP *	1.600	1.236–2.035	<0.001
(Log) Slope max SBP *	1.536	1.241–2.004	0.001
Δ% day–night SBP *	0.683	0.535–0.872	0.002
Sex (M: 1; F = 0)	0.567	0.345–0.932	0.025

* For each one standard deviation increase in the variable.

## Data Availability

The data that support the findings of this study are available from the corresponding author upon reasonable request.
